# Tissue inhibitor of metalloproteinase 1 (TIMP-1) deficiency exacerbates carbon tetrachloride-induced liver injury and fibrosis in mice: involvement of hepatocyte STAT3 in TIMP-1 production

**DOI:** 10.1186/2045-3701-1-14

**Published:** 2011-04-04

**Authors:** Hua Wang, Fouad Lafdil, Lei Wang, Shi Yin, Dechun Feng, Bin Gao

**Affiliations:** 1 Department of Oncology, The Affiliated Provincial Hospital of Anhui Medical University, Hefei, Anhui 230001, PR China; 2 Laboratory of Liver Diseases, National Institute on Alcohol Abuse and Alcoholism, National Institutes of Health, Bethesda, MD 20892, USA; 3 Laboratory of Liver Pathophysiology, INSERM U955, Hopital Henri Mondor, 94010 Creteil Cedex, France

## Abstract

**Background:**

Tissue inhibitor of metalloproteinase 1 (TIMP-1), which is thought to be produced mainly by activated hepatic stellate cells and Kupffer cells in the liver, plays a pivotal role in matrix remodeling during liver injury and repair; while the effect of TIMP-1 on hepatocellular damage remains obscure.

**Results:**

Hepatic expression of TIMP-1 mRNA and protein was up-regulated both in acute and chronic liver injury induced by carbon tetrachloride (CCl_4_). Compared with wild-type mice, TIMP-1 knockout mice were more susceptible to CCl_4_-induced acute and chronic liver injury, as shown by higher levels of serum alanine aminotransferase (ALT), greater number of apoptotic hepatocytes, and more extended necroinflammatory foci. TIMP-1 knockout mice also displayed greater degree of liver fibrosis after chronic CCl_4 _injection when compared with wild-type mice. *In vitro *treatment with TIMP-1 inhibited cycloheximide-induced cell death of primary mouse hepatocytes. Finally, up-regulation of TIMP-1 in the liver and serum after chronic CCl_4 _treatment was markedly diminished in hepatocyte-specific signal transducer and activator of transcription 3 (STAT3) knockout mice. *In vitro *treatment with interleukin-6 stimulated TIMP-1 production in primary mouse hepatocytes, but to a lesser extent in STAT3-deficient hepatocytes.

**Conclusions:**

TIMP-1 plays an important role in protecting against acute and chronic liver injury and subsequently inhibiting liver fibrosis induced by CCl_4_. In addition to activated stellate cells and Kupffer cells, hepatocytes are also responsible for TIMP-1 production during liver injury via a STAT3-dependent manner.

## Introduction

Chronic liver fibrosis induced by viral hepatitis, alcohol abuse, and nonalcoholic steatohepatitis is a major cause of morbidity and mortality worldwide [1]. The progression of liver disease can be defined as an alteration of hepatic parenchyma characterized by two major events: injury and regeneration. The initial cause of the injury determines the loss of hepatocytes including apoptosis and necrosis followed by inflammatory response [2]. Consequently, the loss of tissues or liver injury leads viable hepatocytes to re-enter the cell cycle and divide by mitosis, to replace the lost or damaged hepatocytes [3]. During these wound healing processes, the extracellular matrix (ECM) also undergoes a process of remodeling stimulated by persisting inflammatory injury, which may result in abnormal collagen deposition [1]. This microenvironment alteration responsible for hepatocyte damage and ECM remodeling is highly complex and its mechanisms are not fully understood. It seems that all types of liver cells and a variety of soluble factors are involved in the process of ECM remodeling, contributing to hepatocyte injury, inflammation, fibrosis and liver regeneration [4-8].

Matrix metalloproteinases (MMPs) and their specific inhibitors, the tissue inhibitors of metalloproteinases (TIMPs) play an important role in inducing and preventing the degradation of the ECM, respectively [9]. Many studies have shown that MMPs and TIMPs play a pivotal role in matrix remodeling during hepatic injury and repair [5,8,10-12]. Among them, TIMP-1 is a widely expressed, and secreted protein that plays a critical role in tissue remodeling via inhibiting members of a large family of MMPs [13]. TIMP-1 has been suggested to be a serum marker for liver fibrosis, and the expression is induced during liver injury [14]. In addition, TIMP-1 also plays an important role in promoting liver fibrosis [15-17] but inhibiting liver regeneration [6]. The profibrogenic effects of TIMP-1 are thought to be mediated via preventing collagen degradation through inhibition of MMPs and protecting against activated hepatic stellate cell (HSC) death [17-20]. It is believed that activated HSCs and Kupffer cells are the major sources for TIMP-1 production during liver injury [21]. Although early studies also showed TIMP-1 mRNA and protein expression are up-regulated by inflammatory cytokines in rat hepatocytes [22,23], the precise roles of TIMP-1 produced by hepatocytes in liver injury remain largely unknown. In this study, we found that TIMP-1-deficient (TIMP-1^-/-^) mice were more susceptible to CCl_4_-induced liver injury and fibrosis, suggesting the protective feature of TIMP-1 in liver injury. Moreover, *in vitro *experiments showed that TIMP-1 directly protected against cycloheximide-induced hepatocyte death. Lastly, we provided evidence suggesting that hepatocytes also contribute to TIMP-1 production during chronic liver injury, which is controlled by STAT3.

## Results

### Up-regulation of TIMP-1 in acute and chronic liver injury after CCl_4 _exposure

**Figure 1:**
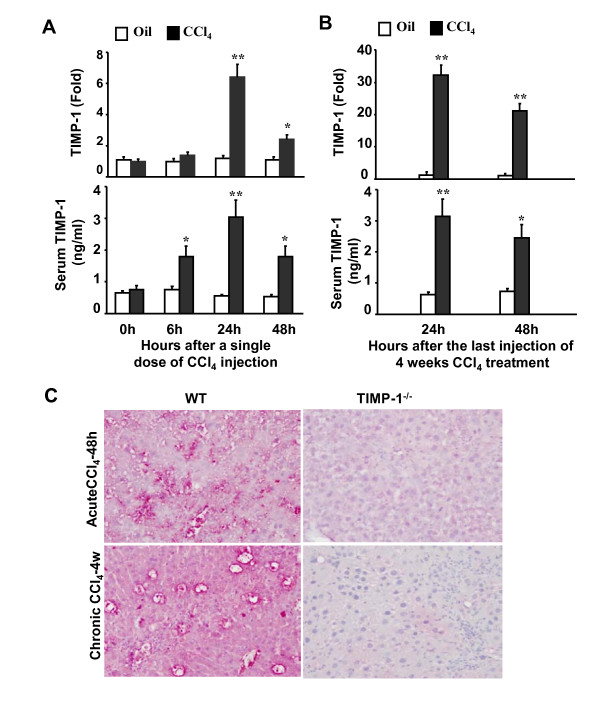
**Up-regulation of hepatic and serum TIMP-1 levels following CCl_4_-induced acute or chronic liver injury in mice**. **A**, Mice were treated with a single dose of CCl_4 _injection or vehicle (olive oil) injection. Liver tissues were then collected and subject to real-time PCR analysis of hepatic TIMP-1 mRNA (top panel). Sera were collected for measurement of TIMP-1 protein by ELISA (low panel). **B**, Mice were treated chronically with CCl_4 _or vehicle (olive oil) for 4 weeks. Liver tissues and sera were collected 24 and 48 h after the last injection and subject to real-time PCR (top panel) and ELISA analyses (low panel) of TIMP-1. The values from vehicle-treated group were set as 1 fold in real-time PCR analyses. Values represent means ± SEM (n = 4). *P < 0.05; **P < 0.01 in comparison with corresponding vehicle-treated groups. **C**, Wild-type (WT) and TIMP-1^-/- ^mice were treated with CCl_4 _for 48 hours or 4 weeks. Liver tissues were collected for immunohistochemical staining with anti-TIMP-1 antibody. Representative pictures are shown (original magnification × 200).

To determine the expression of TIMP-1 during the course of acute and chronic liver injury, real-time PCR and ELISA analyses were performed on liver samples. As shown in Figure 1A, in a murine model of acute liver injury induced by a single dose of CCl_4 _injection, hepatic TIMP-1 mRNA expression was markedly up-regulated with a peak 24 h post CCl_4 _injection. Serum levels of TIMP-1 protein were also significantly elevated after CCl_4 _injection, and were maximal 24 h after injection. Figure 1B shows the hepatic and serum levels of TIMP-1 after a 4-week CCl_4 _treatment. Expression of TIMP-1 mRNA in the liver was about 30 and 20 folds higher at 24 and 48 h following the last CCl_4 _injection, respectively, as compared to corresponding vehicle-treated mice. Similarly, serum levels of TIMP-1 protein were also significantly higher in CCl_4_-treated mice than in control animals. To further determine the source of TIMP-1 production after CCl_4 _challenge, immunohistochemistry staining for TIMP-1 in liver tissue sections were performed. Figure 1C shows that a single injection of CCl_4 _induced markedly TIMP-1 expression in wild-type mice with predominant expression around necroinflammatory areas and weak staining in hepatocytes, while chronic CCl_4 _treatment significantly upregulated expression of TIMP-1 in hepatocytes and nonparenchymal cells. As expected, no TIMP-1 expression was detected in TIMP-1^-/- ^after CCl_4 _injection. These data show that hepatic and serum levels of TIMP-1 are markedly elevated after acute and chronic CCl_4 _treatment.

### TIMP-1^-/- ^mice are more susceptible to acute liver injury induced by CCl_4 _administration

**Figure 2:**
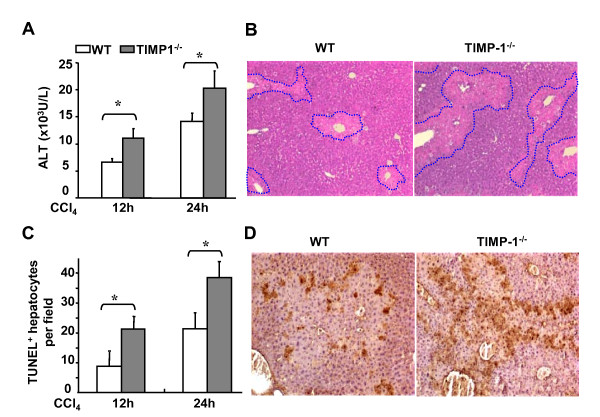
**TIMP-1^-/- ^mice are more susceptible to CCl_4_-induced acute liver injury**. **A, B**, Wild-type and TIMP-1^-/- ^mice were treated with CCl_4 _for 12 or 24 hours. Serum ALT levels were assayed (A). The liver tissue sections were stained with H&E. Representative pictures are shown (original magnification × 100) (B). **C, D**, TUNEL positive hepatocytes were counted (C) and representative pictures of TUNEL staining are shown (D). Values represent means ± SEM (n = 6-10) *P < 0.05.

Although it has been reported that TIMP-1 plays an important role in liver fibrosis and regeneration [6,15-17], its function during hepatocellular injury remains unclear. Figure 2 compared the acute liver injury induced by a single dose of CCl_4 _injection between TIMP-1^-/- ^and wild-type mice. All animals survived after a single dose of CCl_4 _challenge. Figure 2A show that acute injection of CCl_4 _administration induced higher levels of serum ALT and AST in TIMP-1^-/- ^mice than those in wild-type mice. Consistent with serum ALT levels, TIMP-1^-/- ^mice also had larger areas of necrosis than wild-type mice, as assessed by H&E staining (Figure 2B). In addition, acute CCl_4 _administration caused necroinflammatory liver damage with foci located predominantly in pericentral regions in wild-type mice, while TIMP-1^-/- ^mice had a dramatic exacerbation of liver damage with widespread foci of necrotic hepatocytes. Moreover, TUNEL assay shows that TIMP-1^-/- ^mice had a higher number of apoptotic hepatocytes compared with wild-type mice (Figures 2C-D).

### TIMP-1^-/- ^mice are more susceptible to CCl_4_-induce chronic liver injury and fibrosis

**Figure 3:**
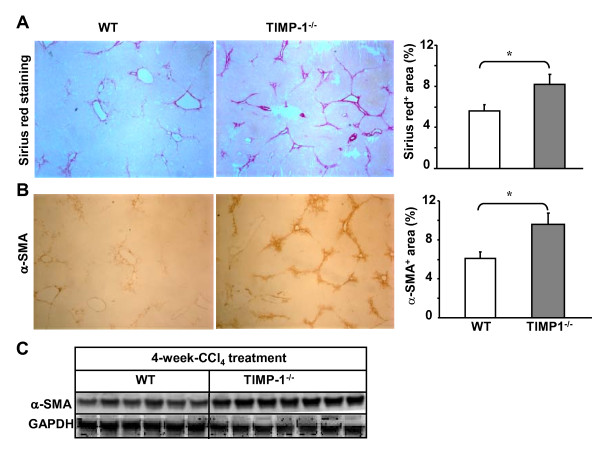
**TIMP-1^-/- ^mice have greater liver fibrosis than wild-type mice post 4-week-CCl4 treatment**. Mice were treated with CCl_4 _for 4 weeks and euthanized 24 hours following the last injection. **A, B**, Liver tissues were collected for Sirius red staining (A) and immunohistochemical staining with α-SMA antibodies (B). The surface areas stained with Sirius red or α-SMA were quantified and shown in the right panel. **C**, Western blot analyses of α-SMA from liver tissue protein extracts. The values represent values ± SEM (n = 5-8). *P < 0.05.

It is well established that activation of HSCs is a key event in the pathophysiology of hepatic fibrosis and is accompanied by induction of TIMP-1 [24,25]. In addition, administration of a TIMP-1 antibody attenuated CCl_4_-induced liver fibrosis [15], thus we hypothesized that deletion of TIMP-1 may reduce liver fibrosis after chronic CCl_4 _exposure. To test this hypothesis, wild-type and TIMP-1^-/- ^mice were treated with CCl_4 _for 4 weeks. Surprisingly, the grade of liver fibrosis was higher in TIMP-1^-/- ^mice with predominant bridging in morphology than that in wild-type mice, which was determined by Sirius red staining for collagen (Figure 3A). Furthermore, immunostaining and Western blot analyses show that expression of α-SMA, a marker for HSC activation, was higher in livers from TIMP-1^-/- ^mice compared with wild-type animals (Figures 3B and 3C).

**Figure 4:**
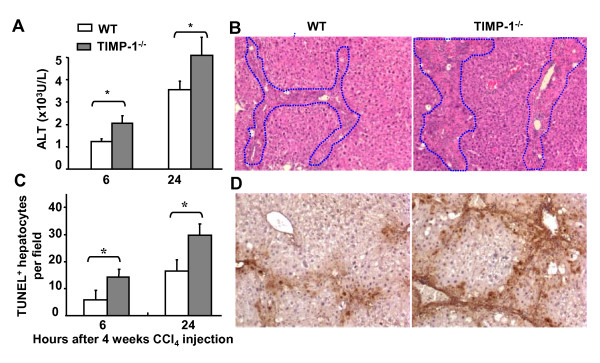
**TIMP-1^-/- ^mice are more susceptible to CCl4-induced chronic liver injury**. Wild-type and TIMP-1^-/- ^mice were treated with CCl_4 _for 4 weeks, and euthanized at various time points after the last injection. **A**, Serum ALT levels were assayed. **B**, The liver sections were stained with H&E and representative pictures are shown (original magnification × 100). **C, D**, TUNEL positive hepatocytes were counted (C) and representative pictures of TUNEL staining are shown (D). *P < 0.05.

To understand the mechanisms underlying the higher liver fibrosis in TIMP-1^-/- ^mice than in wild-type mice, the degree of injury was compared between these two strains of mice after chronic CCl_4 _challenge. In agreement with the greater liver damage in TIMP-1^-/- ^mice after acute CCl_4 _exposure, repeated exposure of TIMP-1^-/- ^mice to CCl_4 _for 4 weeks induced higher levels of ALT, larger area of necrotic hepatocytes, and higher number of apoptotic hepatocytes when compared with those in wild-type mice (Figures 4A-B).

### TIMP-1 directly protects against hepatocyte death *in vitro*

**Figure 5:**
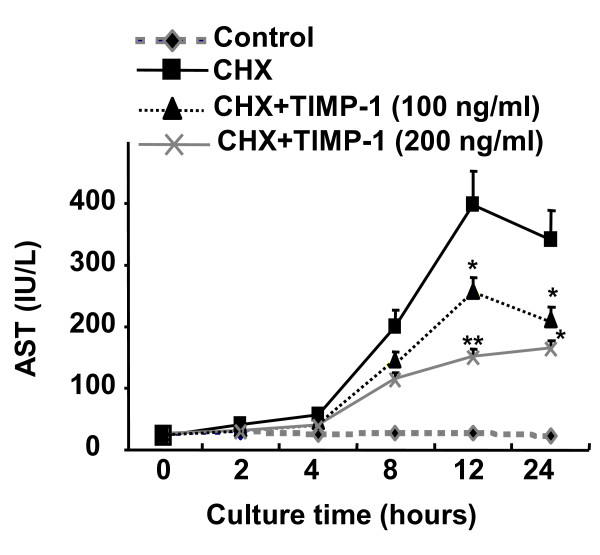
**TIMP-1 is a survival factor for hepatocytes *in vitro***. Hepatocytes (2 × 10^5 ^cells/per well) were cultured in 6-well plates and incubated with or without cycloheximide (CHX) (100 μM) in the absence or presence of TIMP-1(100 ng/ml or 200 ng/ml). Hepatocyte death was determined by measurement of AST levels in the supernatants. *P < 0.05, **P < 0.01 *vs*. corresponding CHX group.

As TIMP-1^-/- ^mice are more susceptible to CCl_4_-induced liver injury, we hypothesized that TIMP-1 may protect against hepatocyte death. We further tested this hypothesis in cultured hepatocytes. As illustrated in Figure 5, incubation of primary hepatocytes with cycloheximide induced hepatocyte cell death as evidenced by an increase in AST release. Pretreatment with TIMP-1 significantly prevented cycloheximide-induced hepatocyte death (Figure 5).

### IL-6 up-regulates TIMP-1 mRNA and protein in primary cultured hepatocytes via a STAT3-dependent manner

**Figure 6:**
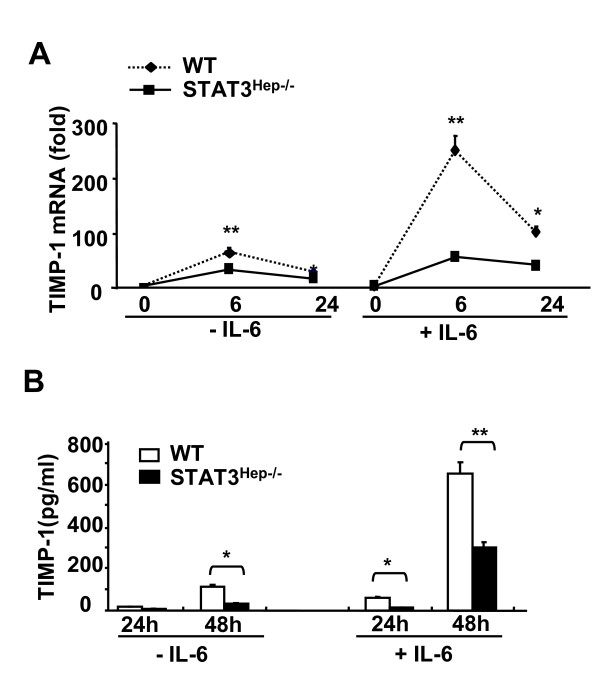
**IL-6 induction of TIMP-1 in primary hepatocytes is mediated via a STAT3-dependent mechanism**. **A**, Hepatocytes (2 × 10^5 ^cells/per well) from wide-type or STAT3^Hep-/- ^mice were cultured in 6-well plates and incubated with or without IL-6 (50 ng/ml) for 6 and 24 h, followed by real-time PCR analysis of TIMP-1 mRNA, **B**, or cultured for 24 and 48 h, followed by collection of the supernatants for measurement of TIMP-1 protein. Values are means ± SE from 4 independent experiments. *P < 0.05, and **P < 0.01 in comparison with the corresponding wild-type groups.

It is believed that TIMP-1 is produced mainly by activated HSCs and Kupffer cells [21], it is not clear whether hepatocytes also contribute to TIMP-1 production. IL-6 has been shown to up-regulate TIMP-1 expression in HSCs and Kupffer cells [26,27]. Here we also demonstrated that treatment of wild-type mouse hepatocytes with IL-6 markedly up-regulated expression of TIMP-1 mRNA hepatocytes, and reaching its highest level at 6 h (Figure 6A). TIMP-1 protein levels in the supernatant were also significantly elevated in IL-6-treated wild-type hepatocytes compared to those without IL-6 treatment (Figure 6B). Moreover, expression of TIMP-1 mRNA (Figure 6A) and protein (Figure 6B) was markedly lower in the hepatocyte from STAT3^Hep-/- ^mice than those from wild-type mice without or with IL-6 treatment.

### Deletion of STAT3 in hepatocytes reduces hepatic and serum levels of TIMP-1 after chronic CCl_4 _treatment

**Figure 7:**
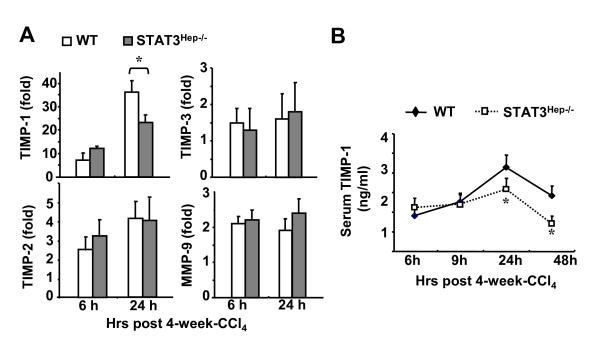
**TIMP-1 production is reduced in STAT3^Hep-/- ^mice during chronic liver injury**. **A**, Mice were treated with CCl_4 _for 4 weeks and euthanized at various time points. Liver tissues were collected and analyzed for TIMP-1, TIMP-2, TIMP-3 and MMP-9. Value from wild-type mice without CCl_4 _treatment was set as 1. **B**, Serum levels of TIMP-1 protein at various time points post 4-week-CCl_4 _treatment. *P < 0.05 in comparison with the corresponding wild-type groups.

To further confirm the critical role of hepatocyte STAT3 in the induction of TIMP-1 during chronic liver injury *in vivo*, we compared the production of TIMP-1 between wild-type and STAT3^Hep-/- ^mice 6 and 24 h after a 4-week chronic CCl_4 _treatment. As shown in Figure 7A, hepatic expression of TIMP-1 mRNA was lower in STAT3^Hep-/- ^mice as compared to wild-type mice 24 h after the last CCl_4 _injection, while the expression of TIMP-2, TIMP-3, and MMP-9 was comparable between these 2 groups. Serum levels of TIMP-1 protein were also lower in STAT3^Hep-/- ^mice 24 h and 48 h post the last injection of CCl_4 _than those in wild-type mice (Figure 7B).

## Discussions

Although the profibrotic effect of TIMP-1 mainly produced by HSCs has been well documented [15,19], we reveal here for the first time an unexpected hepatoprotective feature of TIMP-1 and contribution of hepatocytes to TIMP-1 production during CCl_4_-induced liver injury.

### Hepatoprotection of TIMP-1: dual roles of TIMP-1 in liver fibrosis

TIMP-1^-/- ^mice were more susceptible to hepatocelluar damage induced by CCl_4 _treatment, suggesting that TIMP-1 plays a hepatoprotective role during liver injury. Such hepatoprotection is mediated, at least in part, via directly inhibiting hepatocyte death as TIMP-1 treatment prevented cycloheximide-induced hepatocyte damage (Figure 5). At the present, the mechanism underlying TIMP-1 hepatoprotection remains unknown. TIMP-1 is a survival factor for many cell types dependent and/or independent of the MMP-inhibitory activity [13]. For example, TIMP-1 inhibits HSC apoptosis via MMP inhibition [19], while the anti-apopotic effect of TIMP-1 on human breast carcinoma cells does not require MMP inhibition [28]. Further studies will be required to investigate the mechanism underlying the anti-apoptotic effect of TIMP-1 on hepatocytes.

TIMP-1 has been suggested as a profibrogenic factor to promote liver fibrosis as liver-specific TIMP-1 transgenic mice were resistant to fibrosis resolution [16] and TIMP-1 neutralizing antibody inhibited liver fibrosis [15]. However, surprisingly, TIMP-1^-/- ^mice developed greater fibrosis compared with wild-type mice after CCl_4 _challenge (Figure 3). As TIMP-1 protects against HSC death [19] and hepatocyte apoptosis (Figure 5), we speculate that TIMP-1 may have dual roles in liver fibrosis: stimulating liver fibrosis via promoting HSC survival and inhibiting liver fibrosis via preventing liver injury. The final effect of TIMP-1 on liver fibrosis is determined by the balance between these stimulatory and inhibitory effects. Deletion of TIMP-1 may reduce liver fibrosis through abolishing the profibrogenic effect of TIMP-1, but may also accelerate liver fibrosis by increasing liver injury. Acceleration of liver fibrosis by increased liver injury in TIMP-1^-/- ^mice may dominate over the profibrogenic effect of TIMP-1 on liver fibrosis, leading to greater liver fibrosis in TIMP-1^-/- ^mice after CCl_4 _treatment.

### Hepatocytes contribute to TIMP-1 production during liver injury: controlled by STAT3

Expression of TIMP-1 is induced in the liver during liver injury. It is generally believed that activated HSCs and Kupffer cells are the major source of TIMP-1 production as strong TIMP-1 immunostaining was detected in activated HSCs and Kupffer cells [21]. Our findings here suggest that hepatocytes also contribute significantly to TIMP-1 production that is controlled by STAT3. As shown in Figure 7, serum and hepatic levels of TIMP-1 were lower in STAT3^Hep-/- ^mice, suggesting that activation of STAT3 in hepatocytes plays an important role in induction of TIMP-1 during liver injury. This induction is likely due to the direct stimulation of TIMP-1 production in hepatocytes by STAT3 as *in vitro *IL-6 treatment induced TIMP-1 production in cultured hepatocytes [23,29] and such induction was diminished in STAT3-deficient hepatocytes (Figure 6). In addition, STAT3 binding sites were found on TIMP-1 promoter [30], providing a molecular basis for STAT3-mediated induction of TIMP-1. Finally, the conclusive evidence for contribution of hepatocytes to TIMP-1 production is that TIMP-1 was stained strongly in hepatocytes from the livers of mice with acute and chronic CCl_4 _treatment. Collectively, these findings suggest that in addition to HSCs and Kupffer cells, hepatocytes are also a source for TIMP-1 production which is controlled partially by STAT3 during chronic liver injury.

In summary, our observations collectively identify newly hepatoprotective role of TIMP-1 in a positive feedback manner during liver injury, which is regulated by IL-6/STAT3 signaling pathway. TIMP-1 plays dual roles in regulating liver fibrosis by inhibiting liver fibrosis via protecting against liver injury or by promoting liver fibrosis via protecting against HSC death.

## Materials and methods

### Mice

Eight- to ten-week-old male TIMP-1^-/- ^mice and their wild-type control C57BL/6 mice were purchased from the Jackson laboratory (Bar Harbor, Maine). Hepatocyte-specific STAT3 knockout mice (AlbCre^+/-^STAT3^flox/flox^)(STAT3^Hep-/-^) and their littermate wild-type controls (AlbCre^-^STAT3^flox/flox^) were described previously [31]. All animal experiments were approved by the Institutional Animal Care and Use Committee of the NIAAA.

### CCl_4_-induced liver injury

For acute CCl_4_-induced liver injury, mice were injected (i.p) with a single dose of CCl_4 _(2 ml/kg body weight of 10% CCl_4 _dissolved in olive oil). For chronic CCl_4 _studies, mice received CCl_4 _injection (2 ml/kg body weight of 10% CCl_4_) 3 times a week for up to 4 weeks. Control groups were treated with vehicle (100% olive oil, 2 ml/kg). In chronic studies, the mice were sacrificed at different time points after the last injection of chronic CCl_4 _treatment.

### Blood chemistry

Serum alanine transaminase (ALT) and aspartate aminotransferase (AST) were determined using a chemistry analyzer (PROCHEM-V; Barrow-in-Furness, UK). Serum TIMP-1 levels were assessed by Quantikine enzyme-linked immunosorbent assay (ELISA) kits (R&D Systems, Minneapolis, MN).

### Histological analysis

Formalin-fixed liver samples were processed, and paraffin-embedded liver tissue sections were stained with hematoxylin and eosin (H&E). Liver fibrosis was determined by Sirius Red staining for collagens or immunohistochemical staining for activated HSCs with anti-α-smooth muscle actin (α-SMA) (Dako, Carpinteria, CA), and were quantified by digital imaging with NIH Scion Image and Adobe Photoshop (San Jose, CA). Expression of TIMP-1 in the liver was measured by immunohistochemical staining with anti-TIMP-1 antibody (R&D Systems).

### TUNEL assay

Hepatocyte apoptosis was detected by using an Apoptag Apoptosis Detection Kit (Chemicon International, Temecula, CA) as previously described [32].

### Real Time PCR

Total RNA was purified from about 30 mg liver samples according to the manufacturer (Qiagen, Valencia, CA) and then 1 μg mRNA was reverse-transcribed to cDNA using a High Capacity cDNA Reverse Transcription kit (Invitrogen, Carlsbad, CA). The cDNA template was diluted 1:5 and amplified in real-time PCR using iTaq SYBR Green Supermix (Bio-rad, Hercules CA). An initial denaturation at 95°C for 3 min was followed with PCR cycling: 95°C (15 sec), and 58°C (30 sec) for 40 cycles. Relative mRNA levels were calculated by means of 2^-ΔΔCT ^(ΔΔCT = difference of crossing points of test samples and respective control samples as extracted from amplification curves by the LightCycler software) after normalization to 18S expression used as an internal standard. Fold inductions of analyzed mRNA expression were normalized on 18S RNA expression. The sequences of primers were described previously [31].

### Western blotting

Liver homogenates were prepared in RIPA buffer (50 mM Tris; 1% NP40; 0.25% Deoxycholic acid sodium salt; 150 mM NaCl; 1 mM EGTA) containing 1 mM Na_3_VO_4 _and a protease inhibitor cocktail (Sigma, St. Louis, MO). Protein concentrations were quantified with a detergent compatible protein assay kit (Bio-Rad Laboratories) according to the manufacture's manual. Fifty μg of total protein extracts were denatured in Laemmli buffer containing 5% β-mercaptoethanol, then loaded and separated by gel electrophoresis on a 7% Bis-Tris gel (Invitrogen). Primary antibody was incubated at 4°C overnight under shaking conditions. Immunoreactive bands were visualized on nitrocellulose membranes using alkaline-phosphotase-linked anti-mouse or rabbit antibody and the ECF detection system with a PhosphorImager (GE Healthcare, Piscataway, NJ). Mouse monoclonal anti-α-SMA antibody was obtained from Sigma-Aldrich. Mouse monoclonal anti-GAPDH antibody was obtained from Cell Signaling Technology (Danvers, MA).

### Hepatocyte culture and treatment

Mouse hepatocytes were isolated by *in situ *collagenase perfusion method [31]. Hepatocytes (2 × 10^5 ^cells/per well) were cultured in 6-well plates with serum-free medium and treated with IL-6 (50 ng/ml), followed by the measurement of TIMP-1 protein in culture medium. The hepatocytes were also cultured in medium containing 5% serum and treated with cycloheximide (100 μM) (Sigma) in the presence or absence of recombinant murine TIMP-1 (100 ng/ml or 200 ng/ml). Cycloheximide was used to induce hepatocyte apoptosis. Hepatocyte death was quantified by measuring the activity of released AST in culture medium.

### Statistical Analysis

Data are expressed as means ± SEM (N = 5-12 in each group). Student *t *test was performed to compare values from 2 groups. To compare values obtained from three or more groups, 1-factor analysis of variance (ANOVA) was used, followed by Tukey's post hoc test. Statistical significance was taken at the *P *< 0.05 level.

## List of Abbreviations

ALT: alanine transaminase; AST: aspartate aminotransferase; CCl_4_: carbon tetrachloride; STAT: signal transducer and activator of transcription; STAT3^Hep-/- ^mice: hepatocyte-specific STAT3 knockout mice; MMP: Matrix metalloproteinase; TIMP: tissue inhibitor of metalloproteinase; WT: wild-type mice.

## Competing interests

The authors declare that they have no competing interests.

## Authors' contributions

HW participated in its design, carried out most of experiments, and drafted the manuscript. FL, LW and SY carried CCl_4 _injection, tissue collection, and hepatocyte isolation. DF performed immunohistochemistry analyses of TIMP-1. BG conceived of the study, participated in its design and coordination, and edited the manuscript. All authors read and approved the final manuscript.
